# Efficacy evaluation of acupuncture combined with manipulation for lateral epicondylitis: a meta-analysis

**DOI:** 10.3389/fmed.2026.1892618

**Published:** 2026-08-27

**Authors:** Jing Yin, Guangjun Tang, Baoli Zhao, Yuyang Zhang, Gang Bai, Liyong Liu, Chunli Chi, Wang Hongdong

**Affiliations:** Department of TCM Orthopaedics, Beijing Electric Power Hospital of State Grid Company of China, Beijing, China

**Keywords:** acupuncture, clinical effectiveness, lateral epicondylitis, manipulation, meta-analysis

## Abstract

**Objective:**

To systematically evaluate the efficacy of acupuncture combined with manipulation for relieving pain and improving function in patients with lateral epicondylitis (LE).

**Methods:**

Randomized controlled trials (RCTs) involving patients with LE were retrieved from eight Chinese and English databases from inception to January 1, 2026. The risk of bias of the included studies was assessed using the Cochrane Risk of Bias tool. Meta-analysis was performed using RevMan 5.4.1 software. The main outcomes included clinical effectiveness, visual analog scale (VAS) score, Mayo elbow function score, and pain-free grip strength (PFG).

**Results:**

Fourteen studies met the inclusion criteria. Meta-analysis showed that acupuncture combined with manipulation significantly improved clinical effectiveness (OR = 4.47, 95% CI: 2.8 to 7.14, *p* < 0.00001), Mayo elbow function score (MD = 7.51, 95% CI: 5.98 to 9.05, *p* < 0.00001), and PFG (MD = 2.88, 95% CI: 1.69 to 4.07, *p* < 0.00001). Compared with acupuncture alone (OR = 3.88, 95% CI: 1.95 to 7.73, *p* = 0.0001) or manipulation alone (OR = 4.94, 95% CI: 1.89 to 12.9, *p* = 0.001), the combined intervention was associated with higher clinical effectiveness. Subgroup analyses indicated that acupuncture combined with joint mobilization reduced pain (MD = −1.38, 95% CI: −1.63 to −1.13, *p* < 0.00001). Conventional acupuncture (MD = −0.88, 95% CI: −1.21 to −0.54, *p* < 0.00001) or moxibustion acupuncture (MD = −1.39, 95% CI: −1.65 to −1.12, *p* < 0.00001) combined with manipulation, as well as a 2-week treatment duration (MD = −1.31, 95% CI: −1.55 to −1.08, *p* < 0.00001), was associated with reduced VAS scores. Sensitivity analyses supported robustness of the pooled estimates, whereas funnel plots suggested potential publication bias. The certainty of the evidence was limited by risk of bias and substantial heterogeneity.

**Conclusion:**

Current evidence suggests that acupuncture combined with manipulation may improve clinical effectiveness, elbow function, and pain-free grip strength in patients with lateral epicondylitis. However, the certainty of evidence is low due to high risk of bias and heterogeneity in the included studies. High-quality RCTs are needed to confirm these findings.

**Systematic review registration:**

https://www.crd.york.ac.uk/PROSPERO/, identifier: CRD42024572260.

## 1 Introduction

Lateral epicondylitis (LE), commonly known as tennis elbow because of its frequent occurrence among tennis players, is a common elbow disorder (1). Epidemiological studies suggest that LE affects approximately 1%−3% of the general population, occurs most commonly in individuals aged 30–64 years, and shows no clear sex predominance. The condition can substantially affect daily activities and quality of life (2). LE usually involves the dominant upper limb and is associated with repetitive loading and forceful movement. It typically presents as lateral elbow pain, functional limitation, and pain aggravated by movement (3). Overuse of the elbow muscles and ligaments may lead to injury at the origin of the forearm extensor tendons, most commonly involving the wrist extensor tendons (4, 5). Musculoskeletal ultrasound is widely used for the diagnosis and assessment of LE (6).

Most patients with LE respond to non-surgical treatment, and only approximately 5%−10% require surgical intervention (7). Current non-surgical treatments include corticosteroid injection, oral or topical non-steroidal anti-inflammatory drugs, physical therapy, chiropractic and traditional Chinese medicine (8). However, these approaches may be associated with recurrence, adverse effects, or poor patient tolerance (9). Although individual studies suggest potential benefits of combining acupuncture and manipulation, no previous systematic review has synthesized the evidence on their combined efficacy. Therefore, we conducted this meta-analysis to evaluate whether acupuncture combined with manipulation is superior to acupuncture or manipulation alone for patients with lateral epicondylitis.

## 2 Materials and methods

### 2.1 Study registration and reporting guidelines

The protocol and reporting process of this study followed the Preferred Reporting Items for Systematic Reviews and Meta-Analyses (PRISMA) 2020 statement (10). The protocol was registered in PROSPERO (registration number: CRD42024572260).

### 2.2 Search strategy

A comprehensive literature search was conducted in four English databases and four Chinese databases: PubMed, Embase, the Cochrane Library, Web of Science, China National Knowledge Infrastructure (CNKI), Wanfang Data Knowledge Service Platform (WanFang), VIP Chinese Science and Technology Journals (VIP), and China Biology Medicine (CBM). The search covered the period from database inception to January 1, 2026. No restrictions were imposed on language or publication status. The complete search strategies for all databases are available from the corresponding author upon reasonable request. The retrieval strategy was developed with the assistance of a library and information science expert. For Chinese databases, free-text terms were combined, including “manipulation”, “massage”, “Tuina”, “joint mobilization”, “joint motion”, “kinesitherapy”, “acupuncture”, “tennis elbow”, “lateral humeral epicondylitis”, “lateral epicondylitis”, “RCT”, “randomized controlled trial”, and related terms. For databases supporting controlled vocabulary, such as PubMed/MEDLINE, Embase, and the Cochrane Library, Medical Subject Headings (MeSH), and corresponding title/abstract terms were combined. The PubMed search strategy was adapted as follows:

(((“lateral epicondylitis”[Mesh]) OR “lateral humeral epicondylitis”[Mesh]) AND “LE”[Title/Abstract]) OR (“tennis elbow”[Title/Abstract])) AND ((“Manipulation”[Title/Abstract] OR “massage”[Title/Abstract] OR “Tuina”[Title/Abstract] OR “Joint mobilization”[Title/Abstract] OR “Kinesitherapy”[Title/Abstract] OR “Joint motion”[Title/Abstract])) AND ((“Acupuncture”[Title/Abstract] OR “Electroacupuncture”[Title/Abstract] OR “Dry needle”[Title/Abstract] OR “Fire needle”[Title/Abstract] OR “Floating needle”[Title/Abstract] OR “Surround acupuncture”[Title/Abstract] OR “Intradermal acupuncture”[Title/Abstract])) AND ((“Randomized Controlled Trial”[Publication Type] OR “Randomized Controlled Trial”[Title/Abstract] OR “RCT”[Title/Abstract] OR “Clinical Trial”[Title/Abstract])) AND ((“Efficacy”[Mesh] OR “VAS”[Title/Abstract] OR “Mayo Elbow function score”[Title/Abstract] OR “Pain free grip”[Title/Abstract])).

### 2.3 Inclusion and exclusion criteria

#### 2.3.1 Types of participants

Patients were diagnosed with LE according to explicit diagnostic criteria reported in the original studies, regardless of whether the affected side was left or right.

#### 2.3.2 Types of interventions

The experimental group received acupuncture combined with manipulation, no concomitant use of any medications during the treatment period, treatment frequency of either once daily or every other day. Acupuncture modalities included acupuncture techniques and acupuncture types, the technique included dry needling or surround needling (needles are inserted around the periphery of the lesion site with the tips directed toward the center at approximately 15° to the skin surface, with a spacing of 1.5–2 cm between needles, and one or two additional needles placed at the center) and so on. Acupuncture types included electroacupuncture, fire needle therapy, and other acupuncture-related methods. Manipulation modalities included massage, Tuina, joint mobilization, joint motion, and related manual therapies.

#### 2.3.3 Types of comparisons

Control interventions included acupuncture alone, manipulation alone, no treatment, or other conventional treatments, such as shockwave therapy, oral or topical medication, and local corticosteroid injection.

#### 2.3.4 Types of outcomes

Outcomes included clinical effectiveness, VAS score, Mayo elbow function score, pain-free grip strength of the affected elbow and other relevant indicators.

#### 2.3.5 Types of studies

Only RCTs were included, without restrictions on publication region or country.

#### 2.3.6 Exclusion criteria

Studies were excluded if they were unrelated to the research topic; enrolled patients without LE or with LE combined with other disorders; used interventions other than acupuncture combined with manipulation or included additional combined therapies that could not be separated; were non-RCTs, animal experiments, retrospective studies, reviews, or bibliometric analyses; lacked a comparison group; provided insufficient baseline or endpoint data; contained obvious errors in data or statistical methods; had incomplete content; or were duplicate publications or reports of different follow-up periods from the same trial.

### 2.4 Study screening and data extraction

After completion of the literature search, all records were imported into EndNote X9. Two researchers independently screened the records according to the predefined inclusion and exclusion criteria. Duplicate records and studies that were clearly inconsistent with the research topic were excluded after title and abstract screening. The full texts of the remaining studies were then reviewed, and non-RCTs or studies with statistical errors, incomplete content, or unavailable data were excluded. Disagreements were resolved through discussion; if consensus could not be reached, a third researcher made the final decision.

Two researchers independently extracted data from the included studies and cross-checked the results. Extracted information included the first author, publication year, country, sample size of each group, intervention methods, random sequence generation, allocation concealment, blinding, and outcome data, including clinical effectiveness, VAS score, Mayo elbow function score, and PFG. All extracted data were verified before analysis.

### 2.5 Risk of bias and study quality assessment

The methodological quality of the included studies was evaluated using the Cochrane Collaboration's Risk of Bias tool (RoB 2.0) (11). The following domains were assessed as low, high, or some concerns of bias: randomization process, deviations form the intended interventions, missing outcome data, measurement of the outcome, selection of the reported result and overall assessment. Two researchers independently assessed the included studies and cross-checked the results. Disagreements were first resolved through discussion; if consensus was not reached, a third researcher made the final decision.

### 2.6 Data synthesis

Statistical analyses were performed using RevMan 5.4.1. Heterogeneity was assessed using the Cochrane Q test and the *I*^2^ statistic. When *I*^2^ < = 50%, heterogeneity was considered low or negligible, and a fixed-effect model was used. When *I*^2^ > 50%, sensitivity or subgroup analyses were conducted to explore potential sources of heterogeneity, such as age, disease course, intervention method, or treatment duration. If the source of heterogeneity could not be identified, a random-effects model was used. Effect estimates were reported with 95% confidence intervals (CIs). Odds ratios (ORs) were used for dichotomous variables, and mean differences (MDs) were used for continuous variables. When more than 10 studies were included for an outcome, funnel plots were used to assess potential publication bias (12).

## 3 Results

### 3.1 Literature screen results

**Figure 1:**
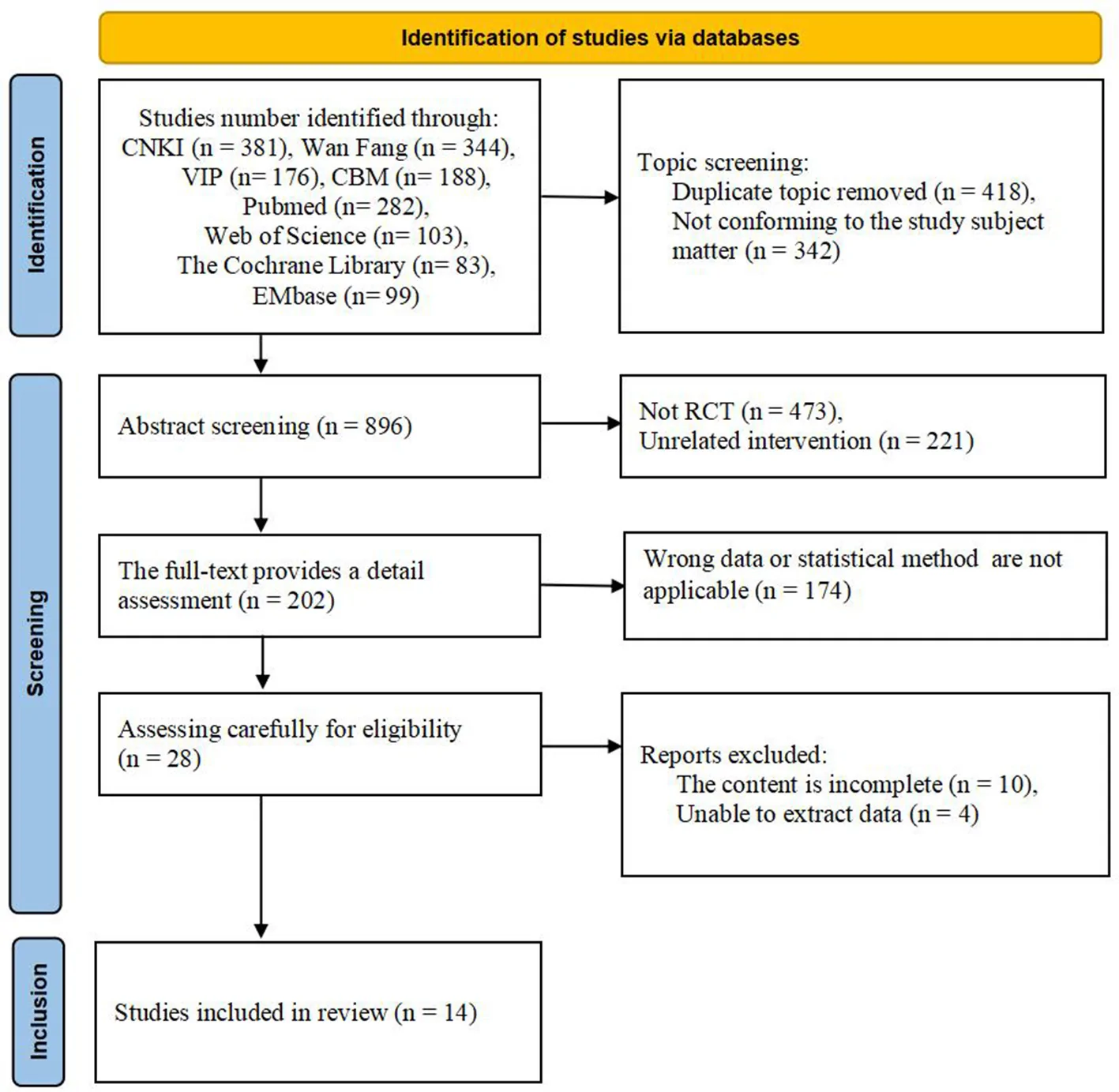
Flow diagram for selection of studies.

A total of 1,656 records were retrieved from the databases. After title and abstract screening and full-text review, 14 studies (13–26) met the inclusion criteria and were included in the analysis. One study was published in English, and the remaining studies were published in Chinese. The literature screening process is shown in Figure 1.

### 3.2 Table of basic characteristics of included studies

Fourteen studies were included in this meta-analysis, involving a total of 959 patients, including 479 patients in the combined-intervention group and 480 patients in the control group. The basic characteristics of the included RCTs are summarized in Table 1. For each study, information is presented on study identification (author and publication year), participant characteristics (sample size in each group), intervention protocols (specific interventions in the experimental and control groups and treatment duration), and primary outcome measures, providing the basis for subsequent analyses and interpretation.

**Table 1:** Basic information of included studies.

References	Sample size		Intervention duration	Intervention measures		Outcome indicators
	Experimental group	Control group		Experimental group	Control group	
Li (13)	32	32	14 days	Fire needling + massage	Acupuncture	I, II, III
Ma et al. (14)	40	40	30 days	Acupuncture + massage	Analgesics external	I
Pei et al. (15)	30	30	25 days	Acupuncture + massage	Acupuncture	I, II
Huang (16)	35	35	14 days	Moxibustion acupuncture + joint motion	Moxibustion acupuncture	I, II
Yang et al. (17)	21	21	14 days	Moxibustion acupuncture + massage	Massage	I, II, III
Ma et al. (18)	30	30	28 days	Acupuncture + joint motion	Joint motion	I, II, III
Chen and Gao (19)	20	20	5 days	Surround acupuncture + massage	Oral analgesics	I, II, III, IV
Hu et al. (20)	29	28	14 days	Acupuncture + joint motion	Needle	I, II, III
Wang et al. (21)	30	30	7 days	Acupuncture + joint motion	Oral analgesics	I, II, III, IV
Yang et al. (22)	31	33	28 days	Acupuncture + massage	Analgesics external	I, II
Zhao and Shao (23)	45	45	28 days	Acupuncture + joint motion	Joint motion	I, III
Li et al. (24)	59	59	25 days	Electroacupuncture + massage	Electric acupuncture	I, II
Liu et al. (25)	40	40	28 days	Surround acupuncture + massage	Surround acupuncture	I, II
Ye (26)	37	37	14 days	Intradermal acupuncture + massage	Massage	I, II, III, IV

I: Clinical effectiveness; II: VAS; III: Mayo elbow function score; IV: Painless grip strength.

### 3.3 Included studies quality assessment

**Figure 2:**
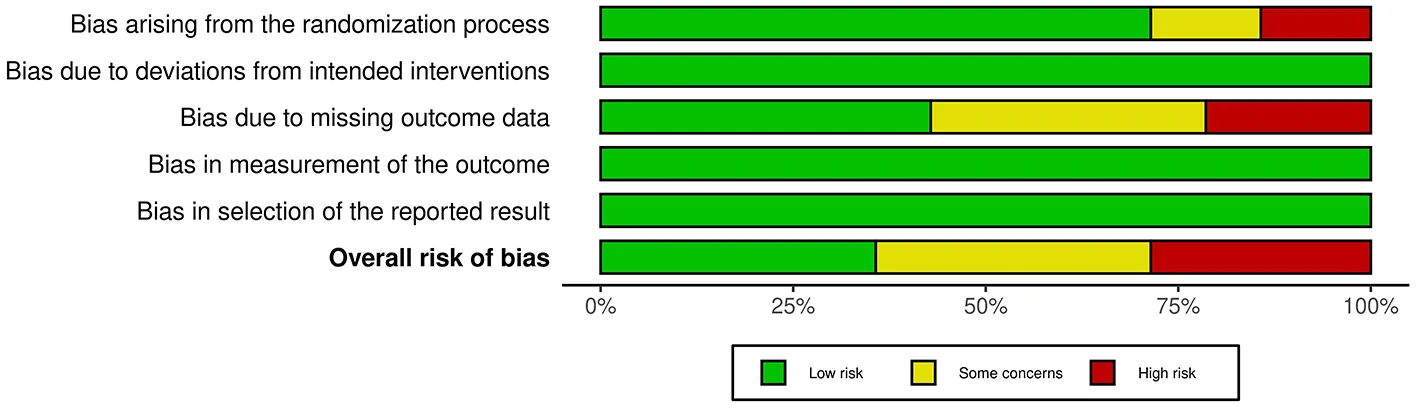
Summary bar chart for bias in the quality of 14 included studies.

**Figure 3:**
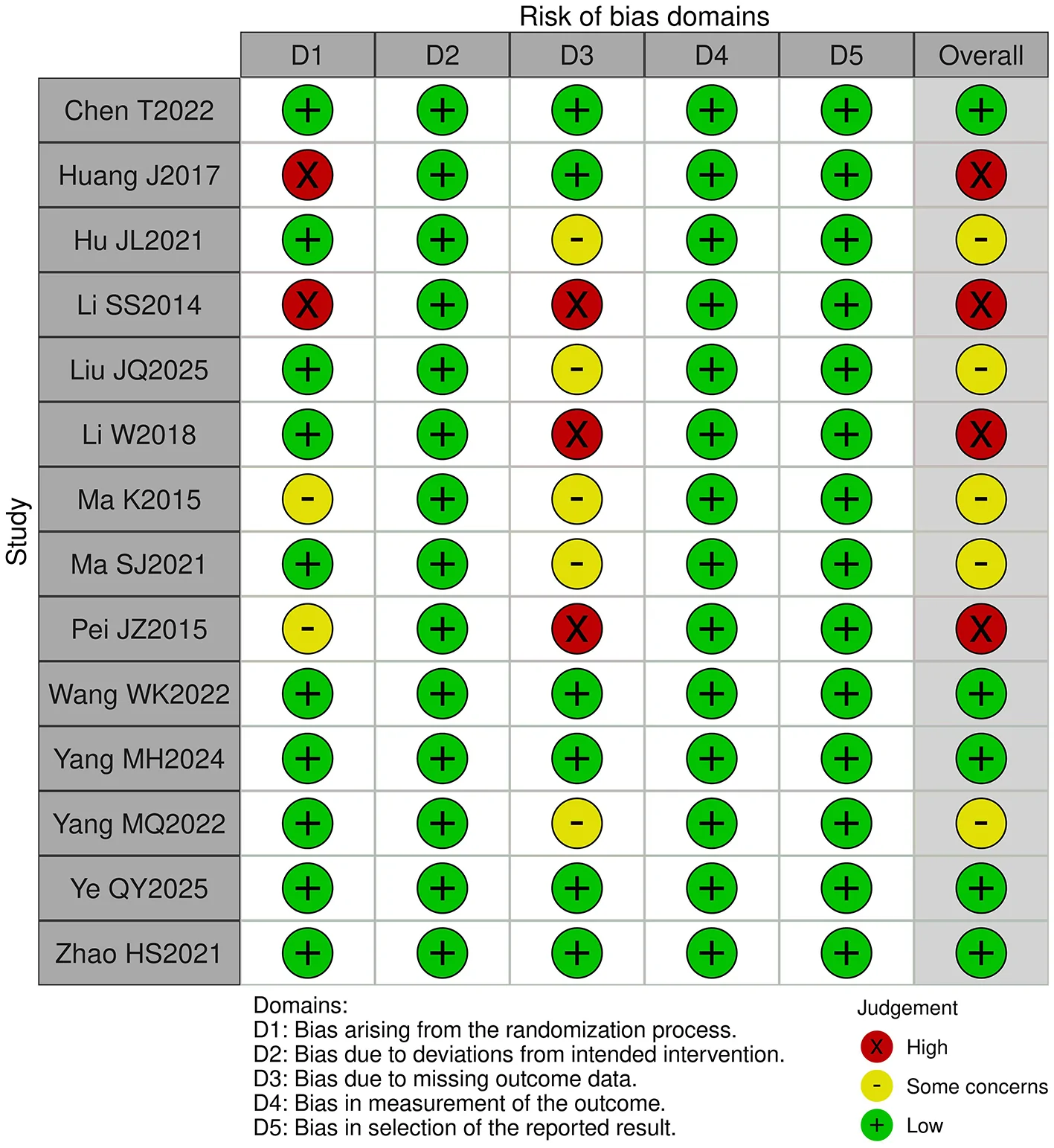
Traffic-light plot of bias risk for the quality of 14 included studies.

The methodological quality assessment results of the 14 included RCTs are presented in Figures 2, 3. For random sequence generation, two studies (16, 24) were rated as having a high risk of bias because inappropriate randomization methods were used, and two studies (14, 15) did not specify the randomization method, resulting in some concerns. Six studies (16, 19, 21–23, 26) reported more than three outcome measures, indicating complete outcome data and a low risk of attrition bias, whereas three studies (13, 15, 24) reported only one or two outcome measures, suggesting incomplete outcome reporting and a high risk of bias. All included studies reported detailed methods for measuring outcome indicators and their statistical analysis results; therefore, both the measurement methods for the outcome indicators and the selection of reported results were classified as low risk.

### 3.4 Meta-analysis results

#### 3.4.1 Clinical effectiveness

**Figure 4:**
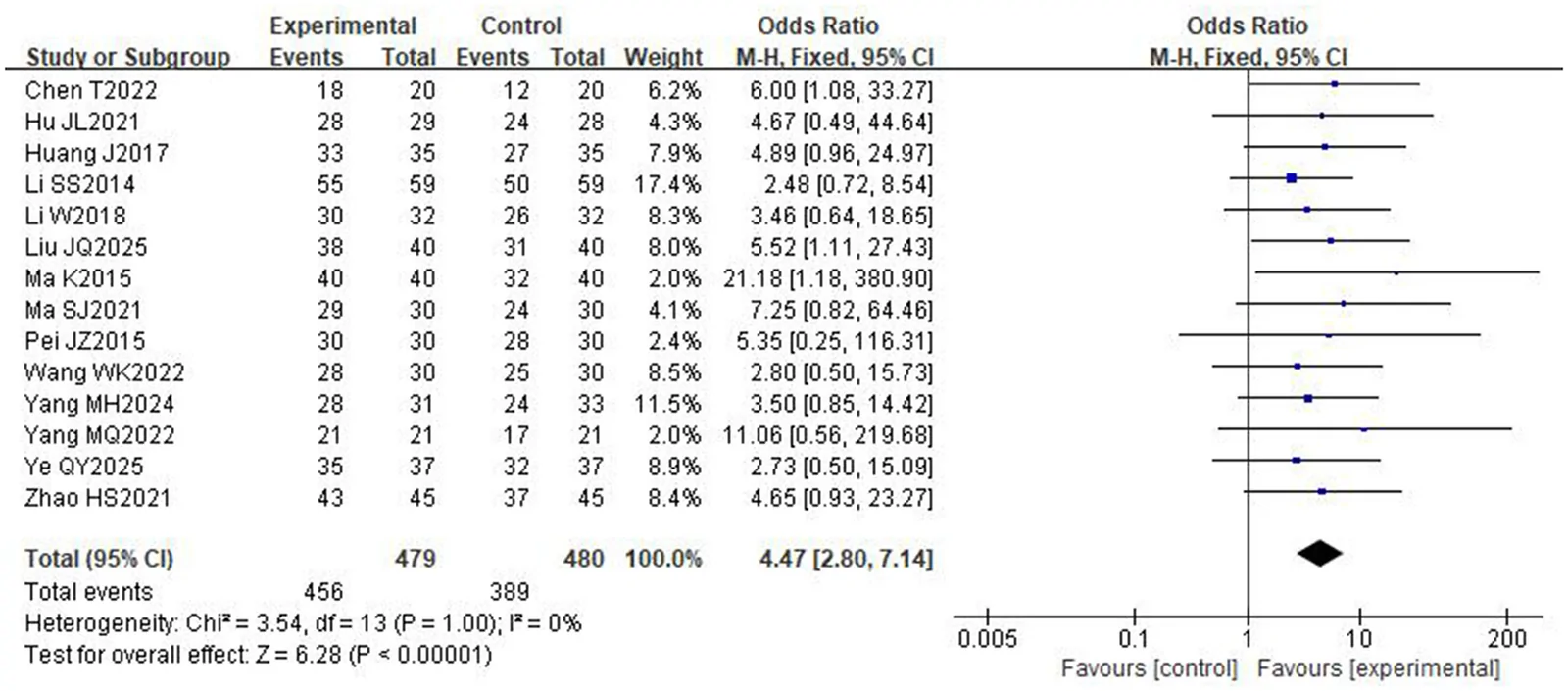
Meta-analysis forest plot of clinical effectiveness.

All included studies reported clinical effectiveness after treatment in the combined-intervention and control groups. The heterogeneity test showed no significant heterogeneity (*p* = 1.00, *I*^2^ = 0%); therefore, a fixed-effect model was used. The analysis showed that acupuncture combined with manipulation had a significantly better effect than the control interventions in patients with LE (OR = 4.47, 95% CI: 2.8 to 7.14, *p* < 0.00001), as shown in Figure 4.

#### 3.4.2 Compare acupuncture used alone

**Figure 5:**
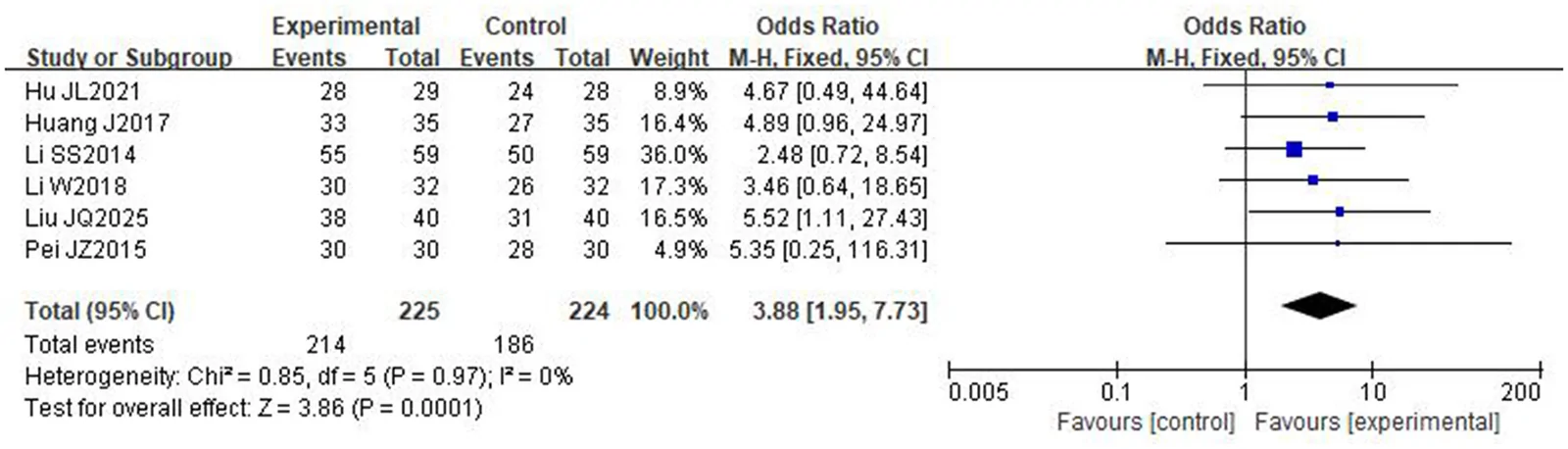
Meta-analysis forest plot of combined group vs. acupuncture alone group.

Six studies reported clinical effectiveness after treatment in the combined-intervention group and the acupuncture-alone group. As shown in Figure 5, heterogeneity was negligible (*p* = 0.97, *I*^2^ = 0%). The fixed-effect model showed that the combined intervention was superior to acupuncture alone (OR = 3.88, 95% CI: 1.95 to 7.73, *p* = 0.0001), with a statistically significant between-group difference.

#### 3.4.3 Compare manipulation used alone

**Figure 6:**
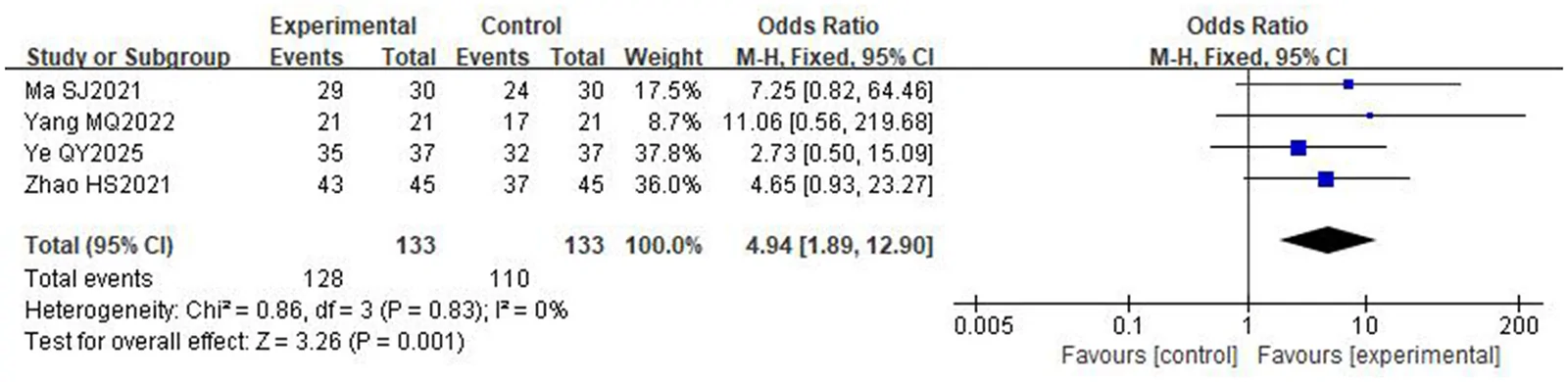
Meta-analysis forest plot of combined group vs. manipulation alone group.

Four studies reported clinical effectiveness after treatment in the combined-intervention group and the manipulation-alone group. As shown in Figure 6, heterogeneity was negligible (*p* = 0.83, *I*^2^ = 0%). The analysis showed that the combined intervention was superior to manipulation alone (OR = 4.94, 95% CI: 1.89 to 12.9, *p* = 0.001), with a statistically significant between-group difference.

#### 3.4.4 VAS score

**Figure 7:**
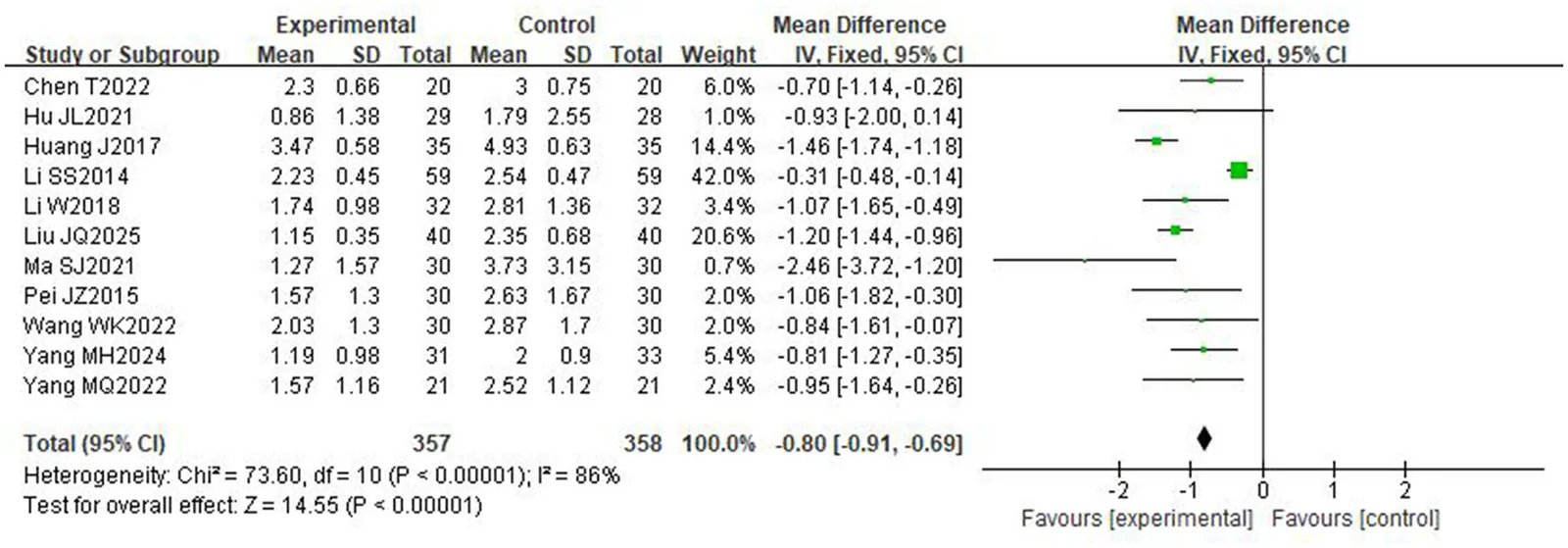
Meta-analysis forest plot of VAS score.

**Figure 8:**
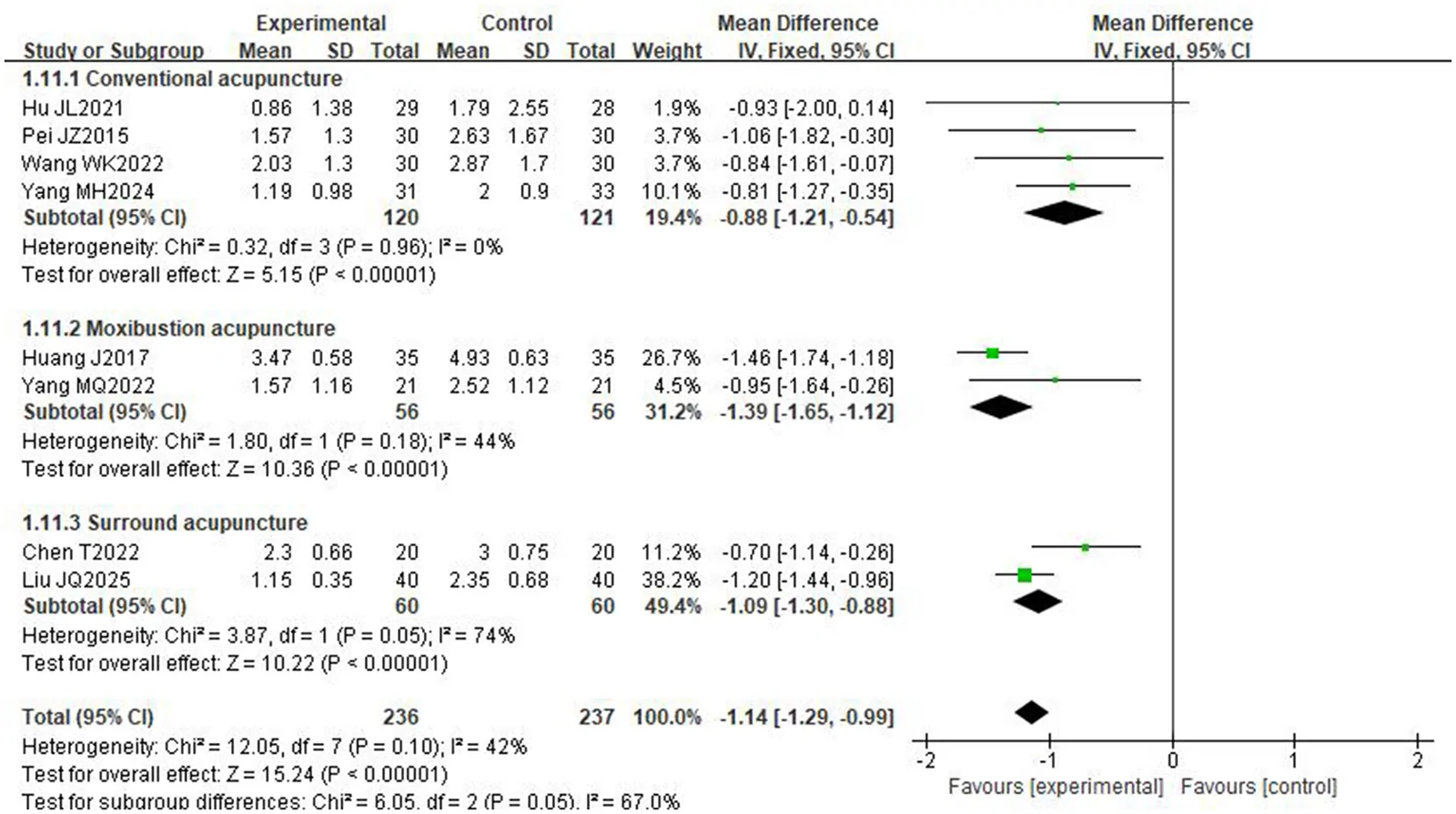
Forest plot of subgroup analysis: acupuncture modality.

**Figure 9:**
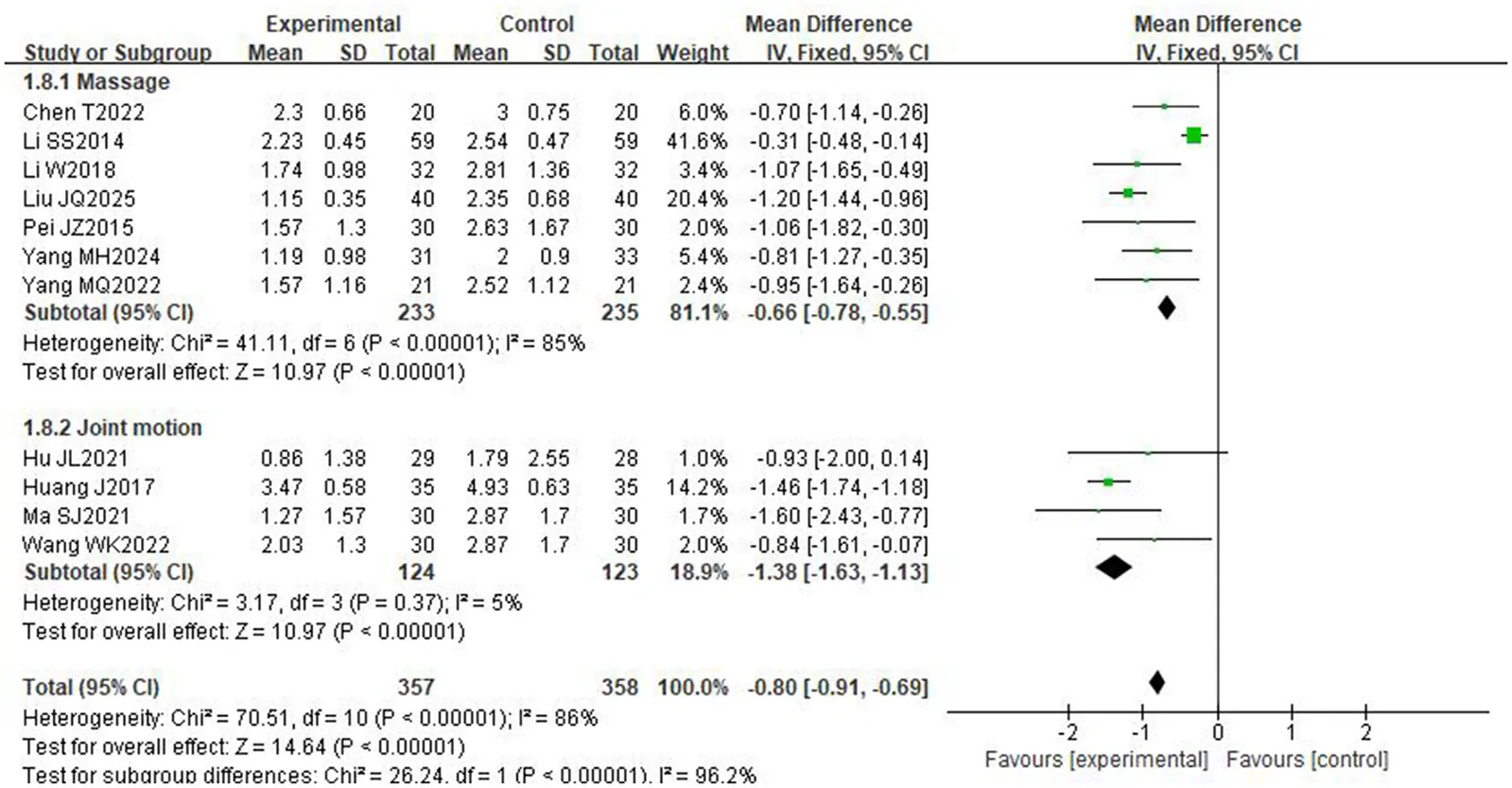
Forest plot of subgroup analysis: manipulation modality.

**Figure 10:**
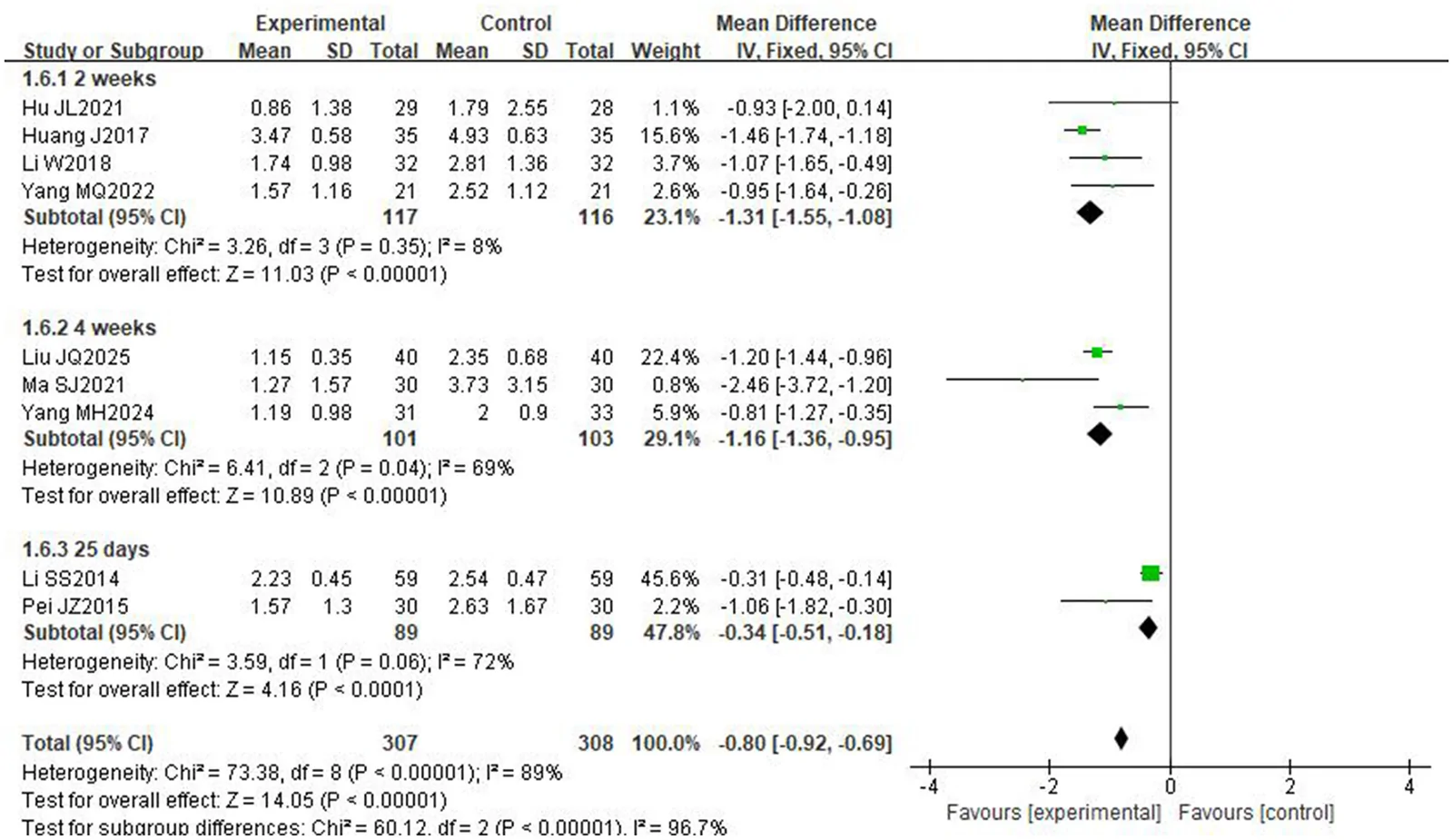
Forest plot of subgroup analysis: intervention duration.

Pain intensity was assessed using the VAS. Twelve studies reported post-treatment VAS scores in the combined-intervention and control groups. After excluding one study (26) with non-estimable data, 11 studies were included in the analysis (Figure 7). The heterogeneity test showed substantial heterogeneity (*p* < 0.00001, *I*^2^ = 86%). Leave-one-out sensitivity analysis did not substantially reduce heterogeneity. Considering differences in acupuncture modality, manipulation technique, and intervention duration among the RCTs, to screen for potential confounding variables that may affect the results of this analysis, subgroup analyses were conducted. As shown in Figure 8, conventional acupuncture combined with manipulation (*I*^2^ = 0%, MD = −0.88, 95% CI: −1.21 to −0.54, *p* < 0.00001) and moxibustion acupuncture combined with manipulation (*I*^2^ = 44%, MD = −1.39, 95% CI: −1.65 to −1.12, *p* < 0.00001) effectively reduced VAS scores in patients with LE. Subgroup analysis by manipulation modality (massage or joint mobilization combined with acupuncture) showed that acupuncture combined with joint mobilization significantly reduced VAS scores (*I*^2^ = 5%, MD = −1.38, 95% CI: −1.63 to −1.13, *p* < 0.00001), as shown in Figure 9. In addition, subgroup analysis by treatment duration indicated that a 2-week course of acupuncture combined with manipulation significantly reduced VAS scores (*I*^2^ = 8%, MD = −1.31, 95% CI: −1.55 to −1.08, *p* < 0.00001), as shown in Figure 10.

#### 3.4.5 Mayo elbow function score

**Figure 11:**
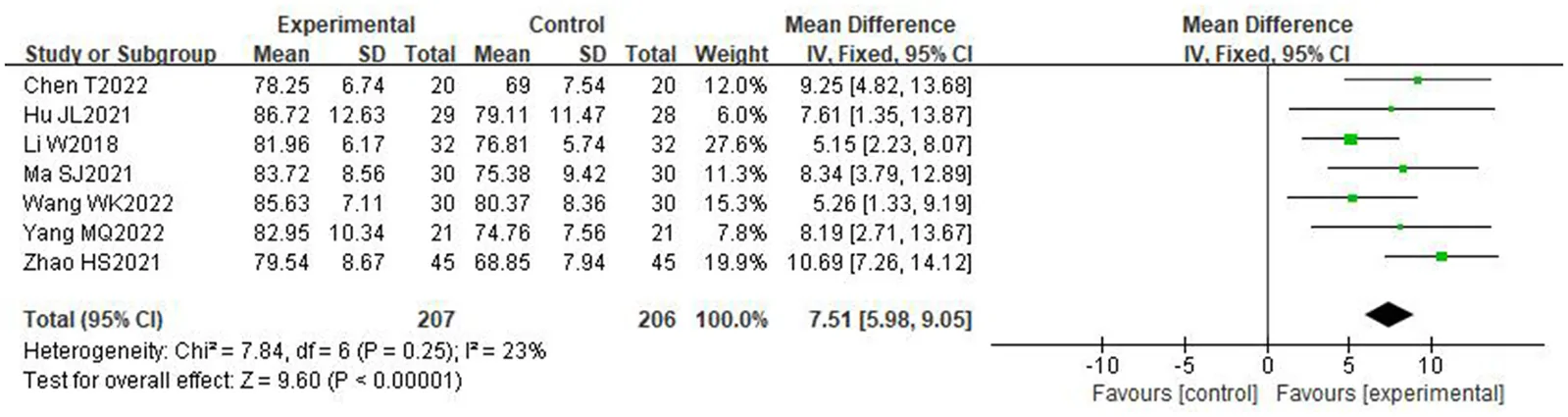
Meta-analysis forest plot of Mayo elbow function score.

Seven studies reported Mayo elbow function scores in the combined-intervention and control groups. As shown in Figure 11, heterogeneity test (*p* = 0.25, *I*^2^ = 23%); therefore, a fixed-effect model was used. The pooled result showed that the combined-intervention group had a significantly greater improvement in Mayo elbow function score than the control group (MD = 7.51, 95% CI: 5.98 to 9.05, *p* < 0.00001).

#### 3.4.6 PFG

**Figure 12:**
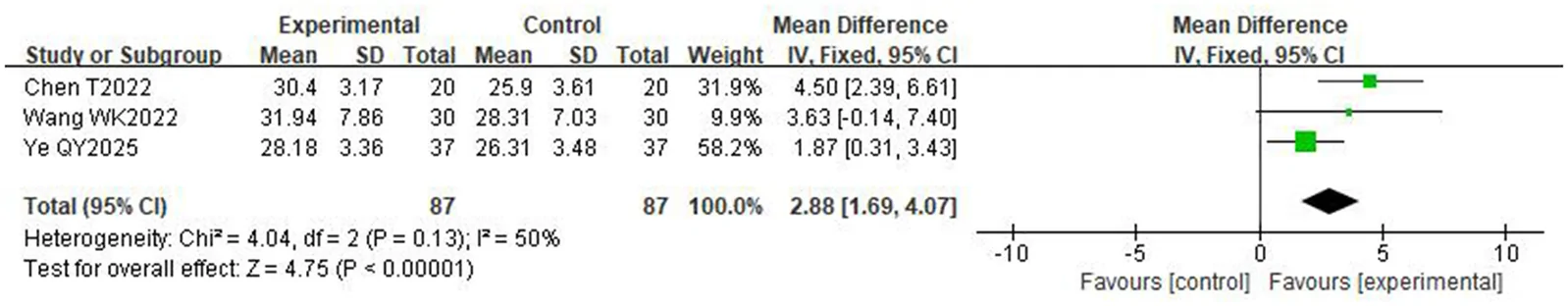
Meta-analysis forest plot of PFG.

Three studies reported post-treatment PFG in the combined-intervention and control groups. The heterogeneity test showed moderate heterogeneity (*p* = 0.13, *I*^2^ = 50%). The pooled result showed that PFG was significantly higher in the combined-intervention group than in the control group (MD = 2.88, 95% CI: 1.69 to 4.07, *p* < 0.00001), as shown in Figure 12.

### 3.5 Sensitivity analysis

Sensitivity analyses were performed for each outcome. Each included study was sequentially removed to assess the stability of the findings. The pooled effect estimates were generally consistent with the original analyses, and no single study substantially altered the overall results, suggesting that the findings were relatively robust.

### 3.6 Publication bias test

**Figure 13:**
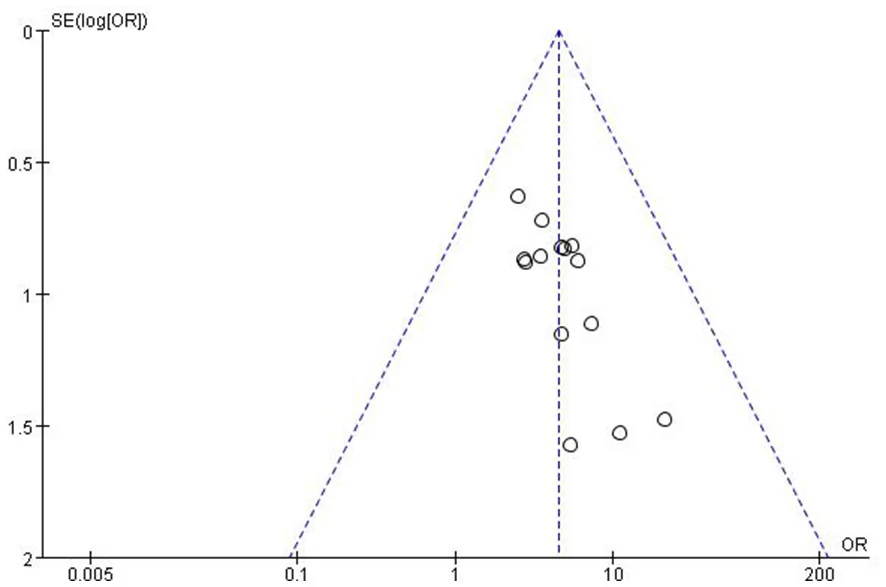
The inverted funnel diagram of clinical efficacy.

Clinical effectiveness was used to assess publication bias because more than 10 studies reported this outcome. As shown in Figure 13, the funnel plot was asymmetric around the pooled effect size (OR = 4.47). Studies with larger effect estimates were more concentrated, whereas studies in the central and right areas of the plot were relatively sparse. This asymmetry suggests the possibility of publication bias, and the high proportion of positive findings among the included studies may have led to overestimation of the intervention effect.

### 3.7 Heterogeneity analysis

Substantial statistical heterogeneity was observed across the 11 included studies for the outcome of pain improvement. To explore potential sources of this heterogeneity, a prespecified subgroup analysis was performed based on the type of manipulation. The test for subgroup differences was statistically significant indicating that the type of manipulation may be a major contributor to the overall heterogeneity. Several factors may account for the high heterogeneity. First, considerable clinical heterogeneity existed in the specific massage techniques employed, with varying treatment durations and frequencies across studies. Second, the control interventions differed substantially, ranging from conventional care to oral medications and physiotherapy, which may have influenced the magnitude of the between-group differences. Third, variations in acupuncture protocols and treatment frequency or courses across studies may have further contributed to the observed inconsistency. In contrast, the low heterogeneity in the joint mobilization subgroup suggests that studies applying this specific manipulation technique yielded more consistent effect estimates, possibly due to more standardized protocols or smaller sample sizes. Taken together, these findings indicate that the overall high heterogeneity is primarily driven by the diversity of massage interventions and study designs, rather than by the efficacy of the combined therapy itself.

## 4 Discussion

### 4.1 Mechanistic insights

LE is a common elbow disorder characterized by aseptic inflammation and degenerative changes of the lateral tendon tissue under excessive mechanical load. It should be differentiated from other conditions that may cause elbow or upper-limb pain, such as periarthritis of the shoulder, elbow fracture, bone tumor, and cervical nerve root lesions (27). Clinical practice guidelines (28) recommend multimodal treatment approaches, including massage and acupuncture, which may produce better therapeutic outcomes.

Manipulation therapy for LE generally includes massage manipulation and joint mobilization. In the included studies, massage manipulation was commonly performed with the patient in a sitting or supine position and the affected arm relaxed. Rolling (gun fa) and kneading (anrou fa) techniques were applied to the lateral elbow and radial forearm for approximately 5 min to relax the extensor muscle group. Subsequently, plucking (tanbo fa) perpendicular to the tendon fibers was performed at the tender point for 1–2 min to release adhesions. Joint mobilization techniques included longitudinal distraction and anteroposterior gliding of the radiohumeral joint. Manual therapy may improve local soft-tissue circulation, reduce compression of small nerve bundles by scar tissue, and increase local tissue temperature to enhance blood flow. Previous reviews (29, 30) suggest that manipulation therapy may be effective for lateral elbow tendinopathy, and RCT evidence (31) has also supported its efficacy in LE.

Acupuncture is one of the most commonly used non-surgical treatments for LE and may promote the repair of injured tissues. In the included studies, acupuncture for LE was administered using disposable stainless-steel needles (0.30 mm x 25–40 mm). The main acupoints included Ashi points (the most tender points around the lateral epicondyle), Quchi (LI11), Shousanli (LI10), Waiguan (TE5), and Hegu (LI4). After routine disinfection, needles were inserted perpendicularly or obliquely to a depth of 10-20 mm until deqi was achieved and were retained for 20–30 min. In studies using electroacupuncture or moxibustion, electrical or warm stimulation was applied at a tolerable intensity for 20–30 min. Acupuncture may mechanically release adhered tendons and fascial tissues, alleviate adhesions and fibrosis caused by chronic strain, dilate local microvessels. Uygur (32) reported that acupuncture was more effective than oral non-steroidal anti-inflammatory drugs (NSAIDs) and proximal forearm braces in improving elbow pain and functional impairment.

In the experimental groups receiving combined therapy, acupuncture and manual therapy were generally administered sequentially during each treatment session, with acupuncture usually performed before manual therapy to achieve analgesia and muscle relaxation. Acupuncture may relieve pain and promote tissue repair through stimulation of specific acupoints, whereas manual therapy may improve joint range of motion and muscle strength through dynamic physical techniques. The combination of these two approaches may therefore exert complementary effects and enhance therapeutic efficacy in patients with LE. However, as this study is a meta-analysis of clinical outcomes rather than a mechanistic investigation, the mechanism are speculative and serve as working hypotheses.

### 4.2 Interpretation of main findings

This meta-analysis of 14 RCTs showed that acupuncture combined with manipulation produced superior clinical outcomes compared with control interventions in patients with LE. The combined intervention was also more effective in improving elbow function and PFG. Compared with acupuncture alone or manipulation alone, the combined intervention significantly improved clinical effectiveness. However, substantial heterogeneity was observed in the pooled analysis of VAS scores, indicating that analgesic effects may be influenced by multiple factors.

Further subgroup analyses helped clarify potential sources of heterogeneity. Based on the included studies, acupuncture modalities were categorized as conventional needling, moxibustion acupuncture, and circumferential needling. The results showed that conventional needling or moxibustion acupuncture combined with manual therapy reduced VAS scores. For manual therapy, subgroup analysis by massage and joint mobilization showed that joint mobilization combined with acupuncture effectively reduced pain. Treatment duration also appeared to be an important moderating factor, and protocols lasting 2 weeks showed significant analgesic effects.

The inverted funnel plot suggested potential publication bias, with positive or statistically significant findings possibly being more likely to be published. This may have led to an overestimation of the true effect size. Because the number of included studies was limited, the funnel plot should be interpreted cautiously. Further large-scale, high-quality, and well-reported RCTs are needed to verify the present findings.

Recent meta-analyses have shown that eccentric exercise (33) and joint mobilization (34) can reduce pain and improve function in patients with LE compared with conventional treatment or no intervention. Another systematic review (35) reported that acupuncture significantly alleviated pain in patients with LE compared with NSAIDs or sham acupuncture. However, previous reviews have generally focused on acupuncture or manipulation alone and have not fully examined the synergistic efficacy of combined treatment modalities. To our knowledge, this is the first meta-analysis to evaluate acupuncture combined with manipulation for LE. The findings suggest that the combined approach may enhance therapeutic outcomes, relieve functional impairment, and improve PFG. Subgroup analyses further indicated that conventional acupuncture or moxibustion acupuncture combined with manipulation may reduce pain, with meaningful relief observed after 2 weeks of treatment. These results support the potential value of multimodal synergistic interventions.

### 4.3 Study limitations

This meta-analysis systematically evaluated RCTs on acupuncture combined with manipulation for LE. Although the included evidence suggests that the combined intervention appears promising, the findings should be interpreted in light of several limitations. First, the sample sizes of the included studies were relatively small, limiting the precision of the estimates. Second, allocation concealment was inadequately reported, and blinding of participants, therapists, and outcome assessors was generally not implemented, which may have affected subjective outcomes such as pain and clinical effectiveness. Third, substantial clinical heterogeneity existed across interventions, including differences in acupuncture methods, manipulation techniques, intervention intensity, treatment frequency, and treatment duration. Inadequate standardization and reporting further reduced the certainty of the conclusions and limited their translation into clinical recommendations. Fourth, all included studies were conducted in China, which may limit the generalizability of the findings and introduce potential selection bias. Fifth, the included studies rarely assessed long-term efficacy, recurrence prevention, or safety outcomes. The lack of internationally recognized functional assessment tools, such as the Patient-Rated Tennis Elbow Evaluation (PRTEE), may also have limited the accuracy and comparability of outcome assessment. Finally, the subgroup analyses were based on predefined hypotheses and should be considered exploratory rather than definitive.

This high risk of bias may have led to an overestimation of the treatment effect in the included trials, This is due to the relatively widespread application of traditional Chinese medicine therapies in China, coupled with the unique characteristics of acupuncture and manipulation treatments, making it impossible to implement a blinded design. Consequently, although our pooled results favor the combined therapy, the magnitude of the benefit should be interpreted with caution, as it may be inflated by methodological shortcomings rather than purely reflecting the true clinical efficacy.

### 4.4 Future prospects

Future studies should use more rigorous trial designs, standardized intervention protocols, and longer follow-up periods to verify the comparative efficacy of different combinations of acupuncture and manipulation for LE and to identify the conditions under which these interventions achieve optimal effects. Multicenter RCTs across different countries and regions are needed to address the current evidence gaps. These trials should follow rigorous methodological standards, use appropriate randomization and allocation concealment, implement blinding whenever feasible, establish long-term follow-up and comprehensive safety monitoring, and include mechanistic sub-studies where appropriate.

## 5 Conclusion

Current evidence suggests that acupuncture combined with manipulation may improve clinical effectiveness, elbow function, and pain-free grip strength in patients with lateral epicondylitis. Conventional acupuncture or moxibustion acupuncture combined with manipulation may effectively reduce pain, with relief observed after 2 weeks of treatment. However, the certainty of evidence is low due to high risk of bias and heterogeneity in the included studies. High-quality RCTs are needed to confirm these findings.

## Data Availability

The original contributions presented in the study are included in the article/supplementary material, further inquiries can be directed to the corresponding author.
